# Editorial: Crossing brain barriers in health and disease: Impact of circadian rhythms

**DOI:** 10.3389/fnins.2023.1176084

**Published:** 2023-03-27

**Authors:** Cecília R. A. Santos, José Cipolla-Neto, Markus Krohn, Isabel Gonçalves, Telma Quintela

**Affiliations:** ^1^CICS-UBI - Health Sciences Research Centre, University of Beira Interior, Covilhã, Portugal; ^2^Department of Physiology and Biophysics, Institute of Biomedical Sciences, University of São Paulo, São Paulo, Brazil; ^3^Immungenetics AG, Hamburg, Germany; ^4^UDI-IPG - Unidade de Investigação para o Desenvolvimento do Interior, Instituto Politécnico da Guarda, Guarda, Portugal

**Keywords:** brain barriers, circadian rhythm, brain disease, circadian therapy, suprachiasmatic nucleus

Brain barriers (BB) are the gatekeepers of the central nervous system, essential to protect the brain from xenobiotics, microorganisms, and other harmful agents. Conversely, disruption of BB is a key event responsible for several changes during the onset and progression of brain diseases (Han and Jiang, 2020).

Along with BB disruption, disturbances in the circadian rhythms are considered a risk factor for Alzheimer's disease and major determinants of many mood, anxiety, and psychotic disorders (Chen et al., 2022).

BB functions are regulated by circadian rhythms (Cuddapah et al., 2019; Quintela et al., 2021), act in concert with neuronal and astrocytic activities (Fultz et al., 2019), and thus hold promise for the improvement of treatments against brain cancer and other brain diseases by disclosing the most adequate timing of the day to enable the brain uptake of therapeutic drugs. On the other hand, neurological disorders have their own circadian patterns in terms of symptoms and responsiveness to therapies (Hood and Amir, 2017).

If dysfunctions of the BB and disturbed circadian rhythms are clear contributors to brain diseases, it follows that circadian-oriented interventions could prevent or delay the onset of brain diseases.

Therefore, this Research Topic gathers different contributions highlighting the involvement of the circadian rhythms in health and disease, contributing to the early identification and control of brain diseases.

It is well-established that disturbances in the circadian rhythms are observed in patients recovering from traumatic brain injury (TBI), which are critical for the recuperation process. The first article of this Research Topic, shows that circadian rhythms are crucial determinants of TBI outcome. The authors showed that health parameters and scores vary depending on the light-dark cycle at which induced TBI occurred, in male Wistar rats (Martinez-Tapiaet al.). Thus, the results of this article will contribute to optimize the medical procedure, taking into account the time of the day when TBI occurs.

The urgent need for new therapies for primary insomnia with minimal side effects, turned out to be the main objective of one of the other articles published. Although the transcutaneous auricular vagus nerve stimulation, has been reported as an effective treatment for insomnia, the inter-individual variations in the efficacy of this treatment are not well-understood. The authors observed that during continuous transcutaneous auricular vagus nerve stimulation, patients with primary insomnia showed variable sensitivity, that might be explained by the distinct control of the sensorimotor network by the autonomic nervous system (Wu et al.).

The link between circadian rhythms and mood and anxiety disorders, was another subject addressed in this Research Topic. The authors analyzed the involvement of the master clock, located in the suprachiasmatic nucleus (SCN) of the hypothalamus, in regulating psychiatric-related behaviors in mice using optogenetic stimulation paradigms. They concluded that SCN-mediated dampening of rhythms is directly correlated with depressive- and anxiety-like behaviors (Vadnie et al.). Thus, this article established an important relationship between the circadian molecular system, namely the SCN, and the psychiatric-related behaviors.

Finally, the last published article also tried to relate alterations in biological rhythms with depressive disorders and consequently possible changes in tumor markers, due to the development of chronic inflammatory diseases. They concluded that the incidence of non-suicidal self-injury, a risk factor for suicide in depressive patients, was relatively high in adolescents. Furthermore, the construction of an index including a combination of depression score, tumor marker levels, and age can identify non-suicidal self-injury behaviors among adolescent patients with diagnosed depressive disorders (Yi et al.).

In summary, this Research Topic is a step forward to understanding the influence of circadian rhythms in health and disease and we hope that the reader will find it a useful reference in the emerging field of adequate “rhythmic” therapeutic strategies taking into consideration the influence of the circadian biology.

## Author contributions

TQ wrote the first draft. CS, JC-N, MK, and IG provided critical comments and editorial suggestions for revisions. All authors agreed on the submitted version.
